# *In vivo versus in vitro* embryo production in small ruminants: strengths, limitations, and practical outcomes

**DOI:** 10.1590/1984-3143-AR2026-0048

**Published:** 2026-07-27

**Authors:** Joanna Maria Gonçalves Souza-Fabjan, Ana Paula Silva Cupello, Nicole Oliveira Rocha, Matheus Silva Admiral Gomes, Marie Saint-Dizier, Ribrio Ivan Tavares Pereira Batista

**Affiliations:** 1 Faculdade de Veterinária, Universidade Federal Fluminense - UFF, Niterói, RJ, Brasil; 2 Institut National de Recherche pour l’agriculture, l’alimentation et l’environnement – INRAE, Centre National de la Recherche Scientifique – CNRS, Université de Tours, Physiologie de la Reproduction et des Comportements, Nouzilly, France; 3 Instituto de Biodiversidade e Sustentabilidade - NUPEM, Universidade Federal do Rio de Janeiro - UFRJ, Macaé, RJ, Brasil

**Keywords:** IVEP, IVF, MOET, goat, sheep

## Abstract

Embryo transfer technologies have become essential tools for accelerating genetic gain, disseminating superior genetics, and preserving valuable germplasm in livestock species. In small ruminants, embryo production has historically relied on multiple ovulation and embryo transfer (MOET), whereas *in vitro* embryo production (IVEP) has expanded substantially over recent decades. Despite this technological progress, the global embryo industry in sheep and goats remains dominated by MOET systems, in contrast to cattle, where IVEP has rapidly become the prevailing technology. This review critically examines the biological, technical, and logistical factors underlying the current balance between MOET and IVEP in small ruminants. We discuss the main biological determinants influencing embryo production efficiency, including oocyte developmental competence, species-specific reproductive traits, and the intrinsic sensitivity of small ruminant gametes and embryos to *in vitro* environments. A comparative analysis with bovine systems highlights that several operational advantages that have driven the expansion of IVEP in cattle, such as the possibility of repeated ovum pick-up without hormonal superstimulation, minimally invasive oocyte recovery, and efficient use of sex-sorted semen, are not readily transferable to sheep and goats. Conversely, recent advances in nonsurgical embryo recovery have reinforced the practical advantages of MOET in small ruminants by reducing procedural invasiveness and improving animal welfare. Emerging perspectives for IVEP are also discussed, including innovations in embryo culture systems and the integration of IVEP with genomic selection and sexed semen technologies. Overall, current evidence indicates that the predominance of MOET in small ruminants reflects not a technological delay but rather a complex interplay of biological robustness and operational simplicity. We believe that IVEP is more likely to expand as a complementary tool rather than to replace MOET as the dominant embryo production strategy in sheep and goats.

## Introduction

Small ruminants play an important role in global livestock systems, contributing to food security, rural livelihoods, and the sustainability of agricultural production in diverse ecological contexts. Sheep and goats are particularly relevant in low-input production systems, where their adaptability to harsh environments and efficiency in converting marginal resources into animal products make them essential for producing meat, milk, wool, and fiber. In this context, reproductive biotechnologies have become increasingly important tools for accelerating genetic progress, disseminating superior genetics, and preserving valuable germplasm. Among those, embryo transfer programs have long relied on multiple ovulation and embryo transfer (MOET) as the principal strategy for embryo production in sheep and goats (Souza-Fabjan et al., 2023a). Over the past decades, however, substantial efforts have been devoted to developing *in vitro* embryo production (IVEP) systems in small ruminants (Souza-Fabjan et al., 2023b), which led to a considerable increase in the use of this technology (Viana, 2025).

The annual statistics compiled by the International Embryo Technology Society (IETS) Data Retrieval Committee represent the most comprehensive and widely recognized source of information on global embryo production and transfer in domestic animals. However, interpretation of these data in small ruminants requires caution, as reporting is voluntary and the number of participating countries varies among years. Consequently, fluctuations in reported embryo numbers may partly reflect differences in reporting coverage rather than actual changes in global production trends. In the most recent report, embryo transfer activity was recorded in 41 countries for cattle, compared with only 11 and seven countries reporting activity in sheep and goats, respectively. In the previous newsletter, however, ovine embryo activity had been reported by 16 countries (Viana, 2024), meaning that five countries were no longer represented in the most recent dataset, which can substantially affect global totals. For example, in that report, the United Kingdom alone recorded 31,449 MOET-derived ovine embryos produced (Viana, 2024), yet no data from this country were included in the most recent IETS newsletter (Viana, 2025) (Table 1). Given that similar levels of commercial activity likely persisted, the absence of data from major contributors can markedly influence global statistics. Therefore, IETS reports should be interpreted primarily as indicators of industry trends rather than as an exact quantification of worldwide embryo production.

**Table 1:** Global trends in ovine and caprine embryo production over the last decade based on International Embryo Technology Society (IETS) Data Retrieval Committee newsletters, including *in vivo* and *in vitro* embryos, leading reporting countries, and the number of countries contributing data each year.

		MOET					IVEP					
**Species**	**Year**	**Countries reporting**	**Donors (nb)**	**Embryos (nb)**	**Embryos/ donor (Mean)**	**Leading country (nb of embryos)**	**Countries reporting**	**Donors (nb)**	**Embryos (nb)**	**Embryos/ donor (Mean)**	**Blastocyst rate (%)**	**Leading country (nb of embryos)**
**Goat**	**2014**	5	260	2,220	8.5	South Africa (1,431)	-	-	-	-	-	-
	**2015**	5	2,079	11,234	5.4	Australia (10,338)	-	-	-	-	-	-
	**2016**	9	465	3,100	6.6	USA (1,586)	-	-	-	-	-	-
	**2017**	5	513	3,975	7.7	USA (3,015)	1	NA	61	-	30.3	USA (61)
	**2018**	7	1,278	8,804	6.8	USA (7,541)	2	26	818	31.4	44.9	South Africa (660)
	**2019**	5	1,144	8,725	7.6	USA (8,725)	2	31	748	24.1	45.9	Spain (748)
	**2020**	7	2,107	13,177	6.2	USA (10,465)	1	383	2,275	5.9	26.7	USA (2,275)
	**2021**	5	1,451	11,193	7.7	USA (8,646)	3	992	6,355	6.4	27.6	USA (6,000)
	**2022**	6	2,192	17,167	7.8	USA (14,605)	2	321	2,297	7.2	33.8	USA (2,147)
	**2023**	6	2,610	15,893	6.1	USA (12,569)	2	1,738	8,568	4.9	21.6	China (5,485)
	**2024**	5	1,728	11,989	6.9	USA (9,932)	2	901	5,312	5.8	27.3	USA (4,731)
**Sheep**	**2014**	8	1,978	14,161	7.1	South Africa (9,132)	-	-	-	-	-	-
	**2015**	15	4,596	32,134	7.0	Australia (15,256)	-	-	-	-	-	-
	**2016**	13	4,951	27,394	5.5	UK (15,499)	-	-	-	-	-	-
	**2017**	9	2,670	18,652	7.0	Brazil (8,816)	1	NA	66	-	33.8	USA (66)
	**2018**	14	2,744	17,353	6.3	Brazil (4,808)	2	50	512	10.2	48.9	Spain (457)
	**2019**	12	3,226	22,374	6.9	Brazil (10,196)	3	NA	1,137	NA	NA	USA (914)
	**2020**	13	4,142	29,819	7.2	Australia (12,427)	1	33	141	4.2	45.3	USA (141)
	**2021**	11	5,781	41,183	7.1	Australia (21,878)	2	174	626	3.5	31.2	USA (446)
	**2022**	10	6,467	42,470	6.5	Australia (22,255)	6	247	704	2.8	27.8	Australia (486)
	**2023**	14	10,637	76,084	7.1	UK (31,449)	5	4,080	20,785	5.0	25.1	China (20,398)
	**2024**	9	6,084	42,394	6.9	USA (19,341)	6	7,805	42,516	5.4	31.4	China (41,248)

MOET: Multiple ovulation and embryo transfer; IVEP: *In vitro* Embryo Production; nb: number; NA: Not available due to insufficient data.

The increase in IVEP in sheep and goats in the most recent report, and the “apparent” reduction (due to unreported data and thus underestimation), reduced the numerical gap between IVEP and MOET compared with previous years. However, interpretation of IVEP activity requires consideration of the geographic distribution of reported data. In the most recent IETS reports, all goat IVEP embryos originated from only two reporting countries, China and the United States, indicating that current IVEP activity in goats is highly concentrated geographically rather than broadly adopted across the global small-ruminant embryo industry. Despite significant technological advances in assisted reproductive technologies, MOET remains the dominant system in small ruminants, whereas adoption of IVEP remains comparatively limited. In contrast, IVEP has expanded rapidly in cattle over the last decade and now dominates the global embryo industry, with more than two million embryos reported worldwide in 2024 alone. This raises an important question: why has the technological trajectory observed in cattle not yet been replicated in sheep and goats?

A simplistic interpretation might suggest that IVEP in small ruminants remains technologically immature. Rather than representing a simple technological competition, the relationship between MOET and IVEP in small ruminants should be understood as a complex interplay among biological efficiency, operational/logistical feasibility, infrastructure requirements, and economic constraints. Thus, the objective of this review is to critically examine MOET and IVEP in small ruminants with respect to biological performance, technical requirements, logistical feasibility, and practical outcomes. We argue that the continued predominance of MOET in sheep and goats reflects the combined influence of biological consistency, operational simplicity, and economic considerations rather than merely a technological delay in IVEP development.

## MOET versus IVEP in small ruminants: insights from bovine systems

**Figure 1:**
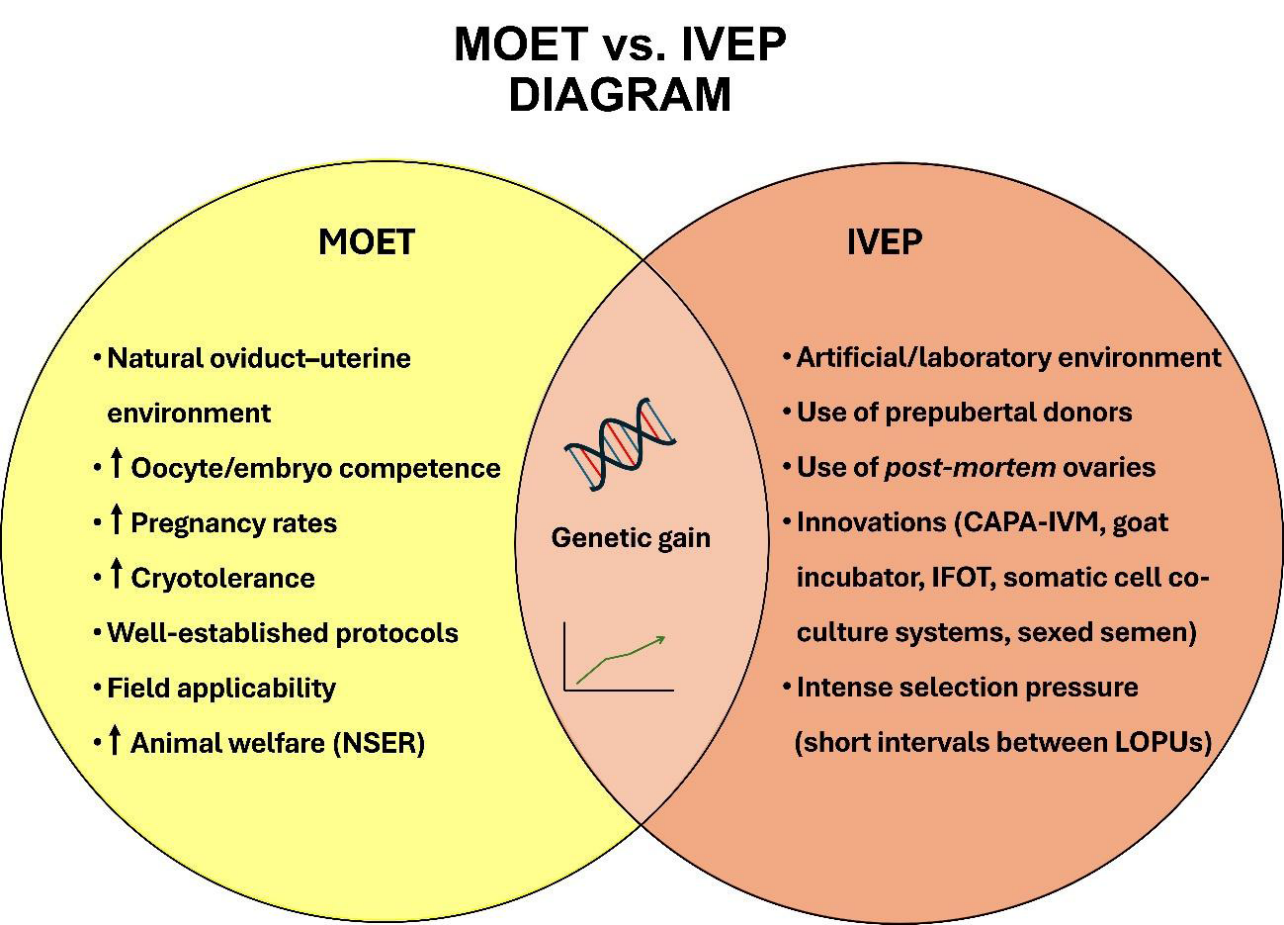
Conceptual comparison between *in vivo* and *in vitro* embryo production strategies in small ruminants. The Venn diagram summarizes key procedures and technological components specific to the MOET and IVEP systems, as well as shared elements between the approaches, illustrating their complementary roles in modern breeding programs. MOET: multiple ovulation and embryo transfer; IVEP: *in vitro* embryo production; LOPU: laparoscopic ovum pick-up; NSER: non-surgical embryo recovery; CAPA-IVM: capacitation *in vitro* maturation; IFOT: intrafollicular oocyte transfer.

A comparison between MOET and IVEP in small ruminants should consider not only embryo output but also biological and logistical factors, including donor physiology, oocyte recovery procedures, and laboratory infrastructure (Table 2; Figure 1). Importantly, many assisted reproductive technologies and culture systems currently used in sheep and goats were initially developed and optimized in cattle. However, small ruminants cannot be considered simply “small cows”, as significant species-specific differences exist in ovarian physiology, follicular dynamics, and reproductive management. In cattle, IVEP efficiency benefits from the possibility of repeated ovum pick-up (OPU) without hormonal superstimulation, because the large antral follicle population supports a continuous pool of recruitable follicles (Bó et al., 2019). In contrast, sheep and goats exhibit smaller follicular populations and consequently lower oocyte yield per collection. While cattle typically present 20–40 recruitable follicles per follicular wave, small ruminants generally recruit only 5–20 follicles, reflecting important differences in ovarian reserve and follicular dynamics (Ginther et al., 1997; Evans et al., 2000; Ireland et al., 2007; Cushman et al., 2009). As a consequence, gonadotropin stimulation is usually required to obtain adequate numbers of oocytes for IVEP in these species (Baldassarre and Karatzas, 2004; Souza-Fabjan et al., 2014a; Baldassarre, 2021).

**Table 2:** Comparative overview of *in vivo* embryo production through MOET (by non-surgical embryo recovery) and *in vitro* embryo production (IVEP) in small ruminants.

**Aspect**	**MOET**	**IVEP**
**Environment or early embryo development**	Natural (oviduct/uterus): greater embryonic competence	Artificial (laboratory): sensitive to *in vitro* environmental conditions
**COC/embryo recovery**	Availability of non-surgical, less invasive, and welfare-enhancing approaches	LOPU surgical procedure, requiring abdominal access (more invasive) and potential COC mechanical damage; Slaughterhouse ovaries: bias (unknown animal history)
**Oocyte maturation**	In the female's physiological environment	*In vitro* environment, high heterogeneity of oocytes, asynchronous maturation, supraphysiological oxygen tension
**Fertilization**	Natural mating or artificial insemination	High incidence of polyspermy; sperm are vulnerable to handling
**Embryo culture**	Oviduct-uterine physiological environments	Static systems: limited to stage-specific demands, greater oxidative stress
**Embryo quality**	Greater: superior cryotolerance and pregnancy rates	Lower: depends on the quality of the oocytes and culture conditions
**Logistics / Equipment requirements**	Simpler / Basic embryo flushing equipment, and a stereomicroscope	Requires a surgical approach and sophisticated laboratory infrastructure
**Need for anesthesia**	Usually mild sedation and local/epidural anesthesia	General anesthesia typically required
**Limitations**	Variability in superovulatory response, limited number of embryos per cycle, and dependence on the efficiency of estrus synchronization	Efficiency varies with the intrinsic quality of the oocytes / Laparoscopic equipment plus full IVEP laboratory infrastructure
**Frequency of repeated collections**	Repeated NSER is possible at relatively short intervals	Repeated LOPU is possible but involves repeated laparoscopic procedures
**Future perspectives**	Remain dominant; employed as a volume-driven technology	Potential for expansion through technical advances: reducing procedural invasiveness and the overall cost per embryo, and improving cryotolerance

MOET: Multiple Ovulation and Embryo Transfer; IVEP: *In vitro* Embryo Production; LOPU: Laparoscopic Ovum Pick-up; COCs: cumulus–oocyte complexes; NSER: Non-surgical Embryo Recovery.

Procedural aspects further influence the relative advantages of these technologies. In cattle, OPU can be performed transvaginally under epidural anesthesia, allowing frequent and minimally invasive oocyte collections. In small ruminants, however, oocyte recovery generally requires laparoscopic ovum pick-up (LOPU), which increases technical complexity and requires specialized equipment (Baldassarre and Karatzas, 2004; Souza-Fabjan et al., 2014a; Baldassarre, 2021). Embryo recovery in MOET programs may also involve invasive procedures, as laparotomy has historically been the most reliable method in sheep, although non-surgical embryo recovery approaches are increasingly being explored (Table 3). Despite these limitations, IVEP offers opportunities that are not readily achievable with MOET. Because oocyte recovery does not depend on ovulation or pregnancy establishment, IVEP can be applied to a broader range of donors, including prepubertal females, aged animals, pregnant donors, and *post-mortem* ovaries, expanding the range of exploitable genetic resources. IVEP also enables the use of laboratory-based technologies such as sex-sorted semen and experimental manipulation of gametes and embryos, although the availability of sexed semen in small ruminants remains limited (Souza-Fabjan et al., 2023a). Conversely, MOET benefits from embryo development *in vivo* within the maternal reproductive tract, which generally supports higher developmental competence and embryo quality compared with *in vitro* systems (Sirard, 2019; Lonergan and Fair, 2016; Coy et al., 2012). Thus, while MOET offers biological advantages through *in vivo* embryo development, IVEP provides greater operational and technological flexibility. Rather than representing competing approaches, both technologies should be viewed as complementary strategies that can be integrated in modern small ruminant breeding programs.

**Table 3:** Comparison between surgical (laparotomy) and nonsurgical (NSER, transcervical) embryo recovery in small ruminants: efficiency, technical requirements, and biological constraints.

**Aspect**	**Laparotomy**	**NSER**
**Invasiveness**	Highly invasive	Minimally invasive
**Anesthesia requirements**	General anesthesia (e.g., ketamine and diazepam mixture or propofol and maintenance with isoflurane) and perioperative analgesia (NSAIDs)	Sedation (e.g., acepromazine), local and epidural anesthesia (lidocaine), analgesia (e.g., dipyrone), and antispasmodic support (n-butyl hyoscine bromide)
**Embryo recovery efficiency**	Reasonably high and consistent under controlled conditions	Variable, influenced by operator skill and animal-related factors
**Repeatability**	Limited due to potential surgical adhesions and recovery time	Allows repeated collections at relatively short intervals
**Recovery time**	Prolonged, requiring postoperative recovery	Short, with rapid return to normal activity
**Risk of complications**	Associated with surgical intervention (e.g., infection, adhesions), depending on surgical and postoperative management. Antibiotics typically required	Generally lower due to the non-surgical nature, although cervical trauma and procedural difficulties may occur depending on the operator's skill
**Animal welfare implications**	May be negatively affected by surgical invasiveness, anesthesia, and recovery period	Generally improved due to reduced invasiveness and faster recovery (although dependent on handling, analgesia, and procedural conditions)
**Field applicability**	Limited, requiring surgical facilities and specialized infrastructure	High
**Operational costs**	Higher due to surgical requirements	Lower to moderate
**Technical requirements**	Surgical expertise, aseptic conditions, and appropriate facilities	Transcervical catheterization skills, appropriate restraint, and operator experience
**Hormonal requirements**	Only standard superovulation protocols	Requires additional hormonal support for cervical relaxation (including PGF2α, estradiol, and oxytocin)
**Influence of breed, age, and parity**	Minimum	Significant, particularly due to cervical complexity in some breeds. Nulliparous usually avoided

α2-agonists: alpha-2 adrenergic agonists; NSAIDs: non-steroidal anti-inflammatory drugs; PGF2α: prostaglandin F2 alpha. References: Fonseca et al. (2019), Menchaca and Hunton (2025), Santos et al. (2020), Souza-Fabjan et al. (2023a).

## Multiple ovulation and embryo transfer (MOET) in small ruminants

**Figure 2:**
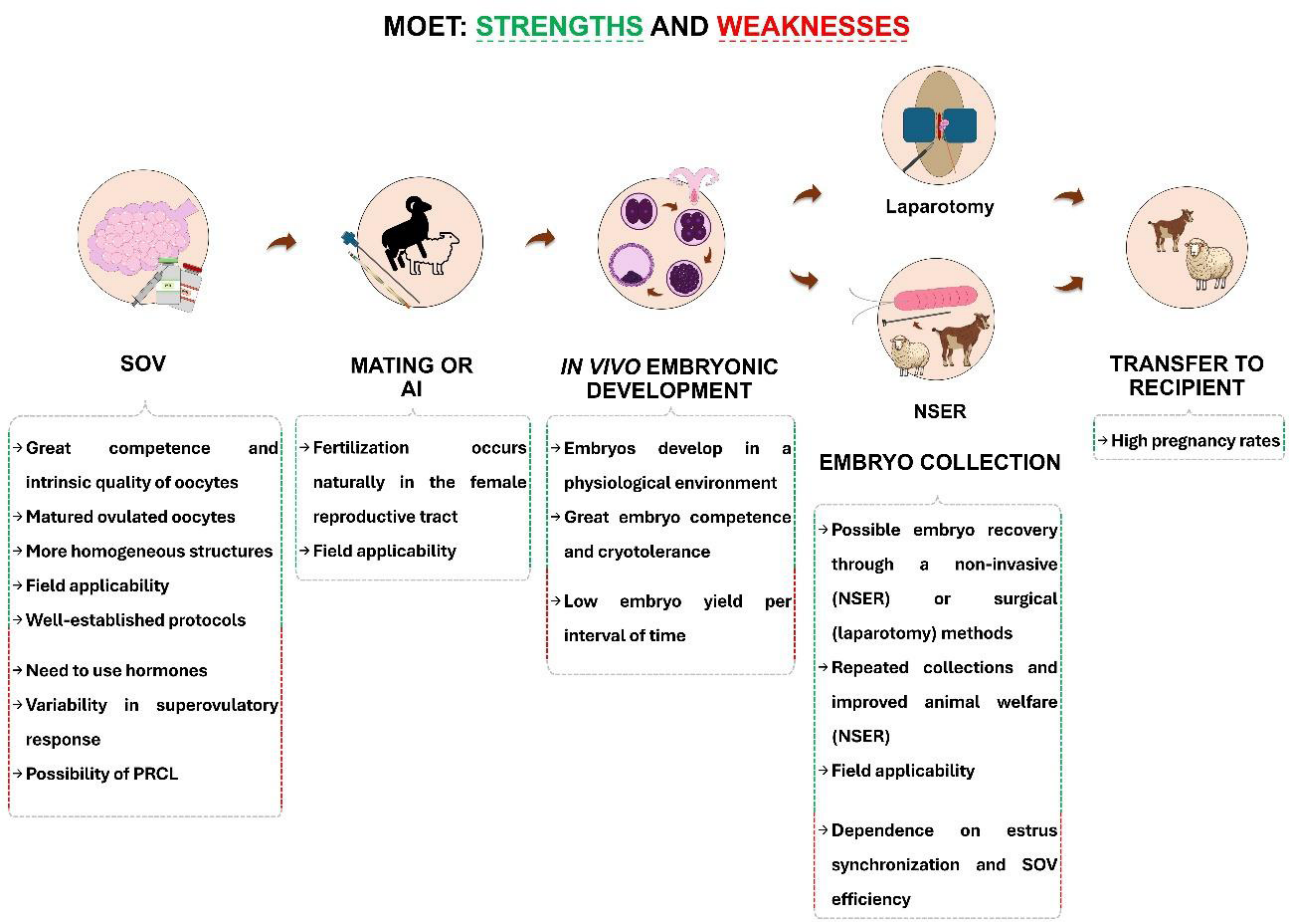
Workflow of *in vivo* embryo production in small ruminants. The scheme illustrates the main procedural steps—from ovarian superstimulation to embryo recovery—and highlights biological and technical strengths and weaknesses that may influence embryo yield and quality at each stage. MOET: multiple ovulation and embryo transfer; SOV: superovulation; PRCL: premature regression of the corpora lutea; AI: artificial insemination; NSER: non-surgical embryo recovery.

*In vivo* embryo production in small ruminants is primarily achieved through MOET. In this technique, donor females are hormonally stimulated to induce the development and ovulation of multiple follicles within a single estrous cycle. Following natural mating or artificial insemination, fertilization and early embryo development occur within the physiological environment of the reproductive tract (Figure 2). Embryos are subsequently recovered from the uterus six to seven days after fertilization and either transferred to synchronized recipient females or cryopreserved for later use (Souza-Fabjan et al., 2023a).

## Biological strengths

*In vivo* embryo production through MOET has long been the predominant method for generating embryos in small ruminants (Paramio and Izquierdo, 2016; Souza-Fabjan et al., 2023a). One of the main biological advantages of this approach is that oocyte maturation, fertilization, and early embryonic development occur within the natural reproductive tract, allowing embryos to develop under physiological conditions in the ovary, oviduct, and uterus. Follicular growth and oocyte maturation occur under tightly regulated endocrine and paracrine signaling pathways, including interactions with granulosa and cumulus cells that support both nuclear and cytoplasmic maturation and the acquisition of developmental competence (Sirard et al., 2006).

After fertilization, the embryo continues its development within the maternal reproductive tract, where it is exposed to a highly regulated microenvironment characterized by dynamic changes in hormones, metabolites, growth factors, extracellular vesicles, and maternal transcripts (Saint-Dizier et al., 2020; Bastos et al., 2022). These components actively modulate embryo gene expression, metabolism, and epigenetic regulation, mediating embryo–maternal communication during the earliest stages of development (Coy et al., 2012; Lonergan and Fair, 2016; Mazzarella et al., 2024). As a result, embryos produced *in vivo* typically display high developmental competence and balanced cellular organization, reflecting the tightly regulated physiological environment in which early development occurs.

Another frequently reported feature of embryos produced through MOET is their superior cryopreservation tolerance and more consistent pregnancy outcomes following embryo transfer. In cattle, pregnancy rates obtained after transfer of *in vitro*-produced embryos are commonly reported to be approximately 20% lower than those achieved with *in vivo*–derived embryos under comparable conditions (Ealy et al., 2019; Lonergan and Fair, 2016; Hansen, 2020). Similar trends have been reported in small ruminants from experimental and field studies. In sheep, comparative studies evaluating different cryopreservation methods demonstrated markedly higher survival rates after transfer for MOET-derived embryos (53.3%) than for IVEP (20.8%) (Dos Santos-Neto et al., 2017). Likewise, in the same study, survival following conventional freezing or vitrification was substantially higher for embryos recovered from superovulated donors than for those produced *in vitro*. Consistent with these findings, pregnancy establishment after embryo transfer has generally been reported to be higher with *in vivo* embryos than with IVP embryos in small ruminants, even though improvements in cryopreservation techniques have progressively reduced these differences (Menchaca et al., 2016; Falchi et al., 2022).

The higher cryotolerance of *in vivo* embryos has been linked to several structural and metabolic characteristics, including lower intracellular lipid accumulation, more stable membrane composition, and more balanced cellular metabolism (Rizos et al., 2008; Banliat et al., 2022). Although substantial progress in culture systems, media formulation, and vitrification techniques has improved the survival of *in vitro*–produced embryos, these features contribute to improved resistance to osmotic and thermal stress during cryopreservation in MOET-embryos. These characteristics help explain why *in vivo*–derived embryos are frequently preferred in programs focused on cryobanking and long-term germplasm preservation (Arrais et al., 2021; Fonseca et al., 2022).

Beyond these aspects, MOET could theoretically offer advantages in sanitary safety and germplasm exchange. According to IETS guidelines, the risk of disease transmission through embryo transfer, regardless of the technique, is very low when standardized sanitary procedures are applied. Most infectious agents do not penetrate the zona pellucida but may attach to its outer surface, which is why sanitary handling protocols focus primarily on decontaminating it before transfer (IETS Manual, Chapter 2). However, structural differences in the zona pellucida between the two types of embryos may also influence pathogen interactions (Vanroose et al., 2000). During early development within the reproductive tract, the zona pellucida undergoes modifications through exposure to oviductal glycoproteins and other maternal secretions, which alter its biochemical composition and surface properties (de la Fuente et al., 2025), whereas embryos produced through IVEP are not exposed to this environment. Experimental studies suggest that such modifications may influence the adhesion of certain pathogens to the IVEP-derived embryo surface, although the biological significance of these observations under field conditions remains uncertain (IETS Manual, Chapter 3).

## Technical and logistical advantages

In small ruminants, embryo recovery can be performed using three main approaches: laparotomy, laparoscopy, or nonsurgical embryo recovery (NSER) (Table 3). Historically, laparotomy was the most widely used technique in these species, largely due to the anatomical complexity of their cervix, which impairs transcervical access to the uterus. Although surgical approaches can provide reliable embryo recovery rates, they are invasive procedures associated with anesthesia, postoperative recovery, and potential surgical complications, which may restrict the frequency with which valuable donors can be used. Over the past decade, however, several research groups, including ours, have focused on developing and refining transcervical embryo recovery techniques. Advances in catheter design, cervical manipulation methods, and hormonal protocols to induce cervical relaxation have progressively improved the feasibility of NSER in both sheep and goats when performed by trained operators (Fonseca et al., 2019; Souza-Fabjan et al., 2023a).

In this aspect, MOET benefits from decades of technical refinement and field application. A practical advantage of MOET systems in small ruminants is the possibility of performing embryo recovery through NSER, a minimally invasive technique that allows repeated collections without the need for surgical intervention. Compared with traditional surgical approaches, NSER reduces animal stress, eliminates the risks associated with abdominal surgery and general anesthesia, and shortens recovery time, thereby improving animal welfare and facilitating the repeated use of valuable donors within breeding programs (Santos et al., 2020; Souza-Fabjan et al., 2022).

Furthermore, NSER can be conducted under field conditions with relatively simple equipment and short procedural times, typically requiring less than 30 minutes per donor when performed by experienced operators. Because the procedure does not require complex surgical infrastructure, it represents a logistically advantageous approach for commercial embryo transfer programs (Figueira et al., 2020; Souza-Fabjan et al., 2023a). The possibility of performing repeated embryo collections at relatively short intervals without major surgical intervention contributes to the operational flexibility and scalability of MOET programs in small ruminants, with no relevant species-specific differences reported between sheep and goats under comparable conditions.

Taken together, these technical and logistical characteristics contribute to the operational simplicity of MOET systems. When efficient NSER protocols are available, *in vivo* embryo production can be implemented with relatively limited laboratory infrastructure and fewer technical steps than *in vitro* embryo production systems.

## Limitations of MOET

One important biological characteristic of MOET programs is the substantial variability observed among donor females in their response to superovulatory treatments. Differences in ovarian follicular dynamics, follicular reserve, endocrine status, genetic background, and physiological conditions can lead to marked variation in the number of ovulations and embryos recovered per treatment cycle (Cognié et al., 2003; Gonzalez-Bulnes et al., 2004; Bartlewski et al., 2016). In sheep and goats, the stage of the follicular wave at the onset of gonadotropin treatment, as well as individual differences in follicle recruitment and dominance patterns, are important determinants of superovulatory response (Bartlewski et al., 2016). As a result, the number of embryos recovered per donor may vary substantially between individuals (0-12; Morais et al., 2025) and across treatment cycles, representing a major biological limitation of MOET programs. Several strategies have therefore been proposed to reduce this variability, including protocols designed to synchronize the emergence of the follicular wave before the initiation of gonadotropin treatment (Gonzalez-Bulnes et al., 2004; Bartlewski et al., 2016). In this context, approaches such as the Day-0 protocol, in which superovulatory treatment is initiated at the time of a newly emerged follicular wave, have been proposed to improve follicular recruitment and enhance the consistency of superovulatory responses in small ruminants (Menchaca et al., 2009; Souza-Fabjan et al., 2017).

In addition to intrinsic biological variability, extrinsic factors associated with hormonal protocols are major determinants of superovulatory response. These include the type and origin of gonadotropins (e.g., pituitary-derived FSH, recombinant FSH, eCG, or their combinations), dosing regimens and administration schedules, as well as synchronization strategies based on progestogens (e.g., CIDR or intravaginal sponges) and luteolytic agents such as prostaglandin F2α or its analogs. Variations in these protocols can markedly influence follicular dynamics, ovulatory response, and embryo yield (Bartlewski et al., 2016; Aybazov et al., 2023; Souza-Fabjan et al., 2023a).

A further limitation affecting the efficiency of MOET programs in small ruminants is the occurrence of premature regression of the corpus luteum (PRCL). This luteal dysfunction results in an early decline in progesterone concentrations, compromising embryo development and frequently reducing embryo recovery and the number of transferable embryos. Studies in both goats and sheep have shown that PRCL may occur in a substantial proportion of superovulated females, with reported incidences ranging from approximately 20% to over 60% depending on the protocol and experimental conditions (Bartlewski et al., 2016; Souza-Fabjan et al., 2017; Rocha et al., 2022). This phenomenon has been associated with endocrine disturbances during superovulation, including elevated estradiol concentrations and the presence of anovulatory follicles, which may trigger premature prostaglandin release and luteolysis. Consequently, PRCL is considered one of the main factors reducing embryo recovery rates and overall efficiency of MOET programs in sheep (Forcada et al., 2011; Rocha et al., 2022). To mitigate PRCL, strategies such as the use of flunixin meglumine (a non-steroidal anti-inflammatory drug inhibiting prostaglandin synthesis) during the early luteal phase have been proposed, leading to improved embryo recovery and production in superovulated ewes (Maia et al., 2024).

Another operational limitation of MOET programs is that embryos recovered from a given superovulatory cycle typically originate from a single male when artificial insemination or natural mating is used. In contrast, IVEP systems allow oocytes to be fertilized with semen from multiple sires within the same session using separate fertilization droplets, increasing flexibility for genetic combinations and enabling more efficient use of semen from elite sires.

## *In vitro* embryo production (IVEP) in small ruminants

**Figure 3:**
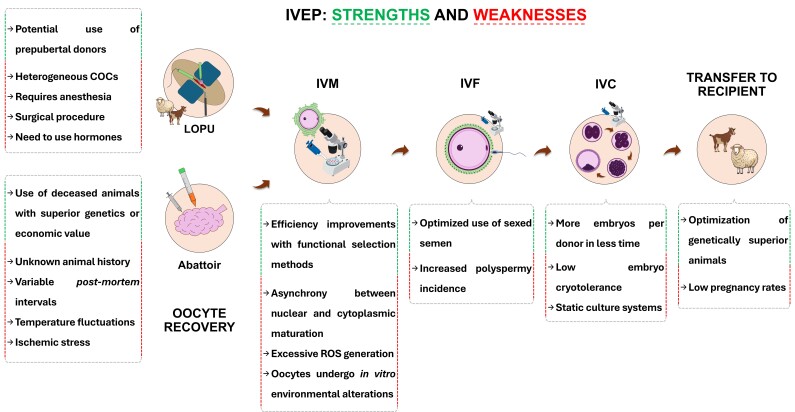
Main steps involved in *in vitro* embryo production in small ruminants, from oocyte recovery to embryo culture. Potential biological and technical strengths and weaknesses affecting developmental competence and embryo quality are indicated along the IVEP pipeline. IVEP: *in vitro* embryo production; LOPU: laparoscopic ovum pick-up; COCs: cumulus–oocyte complexes; IVM: *in vitro* maturation; IVF: *in vitro* fertilization; IVC: *in vitro* culture; ROS: reactive oxygen species.

*In vitro* embryo production, comprising oocyte recovery, *in vitro* maturation (IVM), fertilization (IVF), and embryo culture (IVC), is an important tool for genetic improvement, germplasm conservation, and experimental research in small ruminants (Figure 3). Despite substantial progress, IVEP efficiency in sheep and goats remains lower than that achieved in cattle. This limitation arises from the interaction between species-specific biological characteristics and technical constraints affecting not only oocyte competence, but also sperm selection and quality, fertilization success, and subsequent embryo development. These constraints can be conceptually organized into three interconnected levels: biological, technical, and logistical limitations. Biological factors define the physiological boundaries of follicular development and gamete competence, whereas technical and operational factors influence the efficiency and reproducibility of IVEP systems. Understanding these layers is essential for identifying the main bottlenecks limiting IVEP performance in small ruminants.

## Biological limitations

**Table:** Biological limitations represent intrinsic constraints affecting IVEP efficiency in small ruminants and arise mainly from species-specific characteristics of follicular dynamics, oocyte developmental competence, and early embryonic physiology. A central challenge is the high heterogeneity of oocytes recovered during routine procedures, largely influenced by follicle size, donor age, physiological status, and environmental conditions. This variability directly affects the ability of oocytes to complete maturation, sustain fertilization, and support early embryonic development, thereby defining the biological baseline upon which technical IVEP procedures operate.

### Oocyte developmental competence

Oocyte developmental competence is widely recognized as the primary biological bottleneck limiting IVEP efficiency in small ruminants. Competence refers to the ability of the oocyte to undergo coordinated nuclear and cytoplasmic maturation and subsequently sustain fertilization and early embryonic development (Sirard, 2019). In sheep and goats, oocytes are frequently recovered from follicles at heterogeneous developmental stages, particularly when follicular wave synchronization is not performed, resulting in a large proportion of oocytes with reduced developmental potential (Crozet et al., 1995; Mermillod et al., 1999; Gandolfi et al., 2005; Falchi et al., 2022; Souza-Fabjan et al., 2023b). Oocytes derived from small and medium follicles often exhibit incomplete cytoplasmic maturation, which affects key determinants of competence such as cytoskeletal organization, cortical granule redistribution, and mitochondrial activity (Velilla et al., 2004, 2005, 2006; Anguita et al., 2007, 2008). In contrast, oocytes obtained from larger follicles generally display higher developmental competence, although they represent only a small fraction of recovered oocytes.

In superstimulated ewes, extending the coasting period (the interval between gonadotropin withdrawal and oocyte collection) to 60 h increased the proportion of large follicles and produced oocytes with larger diameters, more advanced germinal vesicle chromatin condensation, and higher expression of maternal-effect and cumulus–oocyte communication genes, despite no detectable differences in conventional morphological or metabolic selection criteria (Pinheiro et al., 2023). These observations highlight that the developmental potential of oocytes entering IVEP systems is strongly conditioned by their follicular history, emphasizing the importance of physiological factors influencing follicular development.

### Physiological modulators of IVEP efficiency

Beyond intrinsic oocyte competence, several physiological factors influence IVEP efficiency in small ruminants by affecting follicular dynamics and the follicular microenvironment. Reproductive seasonality is a major determinant, as photoperiod-driven changes in ovarian activity influence follicular development and endocrine support, resulting in higher embryo development during the breeding season and reduced outcomes during seasonal anestrus (Tibary et al., 2005; Souza-Fabjan et al., 2021). However, in lower-latitude regions, such as the tropical and subtropical areas of South America, including Brazil, seasonal effects may be less pronounced due to reduced photoperiod variation and the use of management strategies that mitigate reproductive seasonality. Donor age also affects developmental competence, as oocytes from prepubertal females show reduced potential due to incomplete nuclear and cytoplasmic maturation (Paramio and Izquierdo, 2014, 2016; Souza-Fabjan et al., 2023b). Nutritional and metabolic status further modulate oocyte quality through their effects on endocrine balance and follicular fluid composition (Diskin et al., 2003; Leroy et al., 2004). These physiological factors (including season, age, and nutrition) also influence the response to ovarian superstimulation and, consequently, affect the outcomes of MOET. However, their impact may be more pronounced in IVEP systems because oocytes must undergo IVM, making their developmental competence more dependent on their initial physiological condition. In addition, genetic and breed-related variability influence ovarian reserve, follicular dynamics, and reproductive patterns, ultimately affecting oocyte yield and developmental competence in IVEP systems (Cognié et al., 2004; Mermillod et al., 2008). Together, these physiological factors contribute to the variability observed in embryo production outcomes in small ruminants.

### Sensitivity to in vitro environments

**Figure 4:**
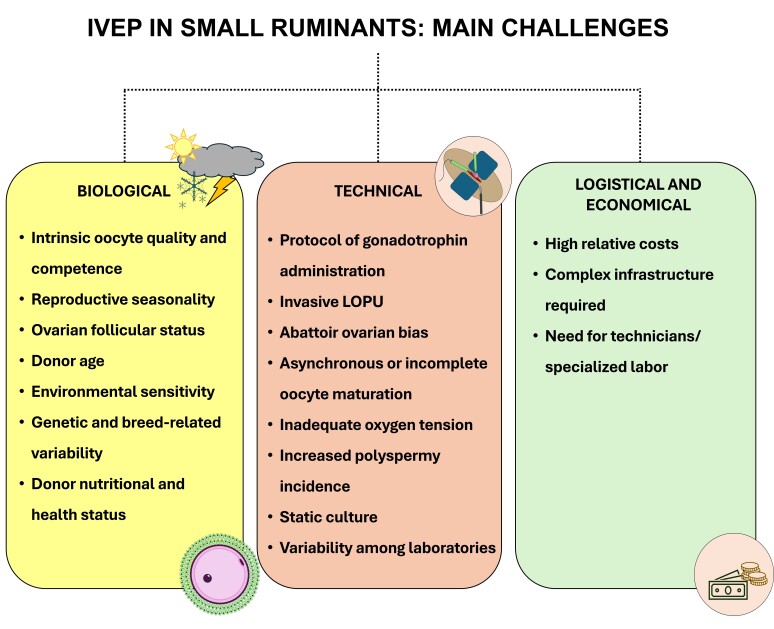
Overview of the major constraints limiting *in vitro* embryo production efficiency in small ruminants. Challenges are organized into distinct interacting domains—biological, technical, logistical, and economical—that illustrate the multifactorial nature of the limitations affecting the scalability and field application of IVEP systems. IVEP: *in vitro* embryo production; LOPU: laparoscopic ovum pick-up.

In addition to intrinsic competence and physiological background, gametes and embryos from ovine and caprine species exhibit increased sensitivity to *in vitro* environments compared with bovine embryos and with embryos derived *in vivo*. However, this apparent sensitivity may not solely reflect intrinsic biological differences, but also the widespread use of *in vitro* culture systems originally optimized for bovine embryos, which may not fully meet the specific physiological and metabolic requirements of small ruminants (Souza-Fabjan et al., 2023a). Ovine and caprine oocytes and embryos show limited tolerance to variations in oxygen tension, redox balance, and metabolic conditions (Rodríguez-Dorta et al., 2007; Natarajan et al., 2010; Amiridis and Cseh, 2012). This vulnerability is partly associated with limited antioxidant defenses and metabolic buffering capacity, increasing susceptibility to reactive oxygen species (ROS), mitochondrial dysfunction, and redox imbalance (Rizos et al., 2002; Mauchart et al., 2023). As a consequence, developmental arrest frequently occurs during critical stages of early embryogenesis, particularly around embryonic genome activation and during the transition from the 8-cell stage to the morula. Importantly, environmental sensitivity interacts with intrinsic oocyte competence and follicular background. Oocytes with incomplete cytoplasmic maturation exhibit reduced tolerance to oxidative and metabolic stress, thereby amplifying developmental losses during IVEP procedures. Together, intrinsic competence, physiological modulation of follicular development, and sensitivity to *in vitro* environments (both biological and system-derived) define the framework that limits IVEP efficiency in small ruminants and ultimately shapes the outcomes of subsequent technical procedures (Figure 4).

## Technical limitations in IVEP

Beyond intrinsic biological constraints, the efficiency and reproducibility of IVEP in small ruminants are also influenced by technical limitations inherent to current laboratory systems. Importantly, many of these limitations reflect incomplete knowledge of the biological dynamics regulating gamete maturation, fertilization, and early embryonic development in these species. As a result, IVM, IVF, and IVC systems remain unable to fully reproduce the dynamic ovarian, oviductal, and uterine environments that coordinate these processes *in vivo*. This limitation is particularly evident for the oviduct, whose microenvironment plays a critical role in sperm capacitation, gamete interaction, and early embryo development. Despite its importance, detailed information on the biochemical composition and temporal dynamics of the oviductal environment in sheep and goats remains scarce, making it difficult to accurately mimic these conditions *in vitro*. Consequently, several aspects of current IVEP systems are simplified approximations of complex physiological processes, contributing to reduced efficiency and variability in embryo production in small ruminants.

### Oocyte recovery: LOPU and abattoir-derived ovaries

Oocyte recovery represents a major technical bottleneck in small ruminant IVEP, as collection methods directly influence oocyte yield, integrity, and developmental competence. Laparoscopic ovum pick-up is indispensable for recovering oocytes from live donors but is invasive and technically demanding, requiring specialized equipment, trained personnel, and careful animal management, which limits collection frequency and scalability (Souza-Fabjan et al., 2014a; Baldassarre, 2021). Procedural parameters such as aspiration pressure, needle gauge, and operator proficiency can affect cumulus–oocyte complex (COC) integrity, with suboptimal settings causing mechanical damage and reduced *in vitro* performance (Wieczorek et al., 2020; Souza-Fabjan et al., 2014a; Baldassarre et al., 2014). In addition, repeated ovarian manipulation during frequent LOPU sessions may induce local tissue stress and inflammatory responses, compromising recovery efficiency and embryo development (Teixeira et al., 2011).

Abattoir-derived ovaries are widely used for methodological optimization due to their availability, low cost, and high oocyte throughput (Ebrahimi et al., 2025). This approach has supported the development of IVM, IVF, and IVC systems under controlled laboratory conditions (Souza-Fabjan et al., 2023b), but introduces technical bias because oocytes originate from donors of unknown age, metabolic and reproductive status and are exposed to variable *post-mortem* conditions before processing (Paramio and Izquierdo, 2014). Such factors can impair oocyte quality, mitochondrial function, and redox balance (Lonergan and Fair, 2014; Reader et al., 2017). Consequently, IVEP systems optimized with slaughterhouse-derived oocytes may overestimate robustness and perform inconsistently when applied to LOPU-derived oocytes from live donors, reinforcing oocyte recovery as a key technical limitation affecting IVEP reproducibility in sheep and goats (Souza-Fabjan et al., 2014b).

### Oocyte selection and in vitro maturation (IVM)

Oocyte selection represents the first technical filter in small ruminant IVEP, as the developmental competence of the oocyte entering IVM largely determines subsequent embryonic outcomes. Morphological evaluation based on cumulus investment and cytoplasmic appearance remains the most commonly used approach; however, it is subjective and only weakly predictive of true developmental competence (Ebrahimi et al., 2025). Functional selection methods, such as brilliant cresyl blue (BCB) staining, which reflects glucose-6-phosphate dehydrogenase (G6PDH) activity and cytoplasmic maturity, have therefore been proposed as more biologically informative indicators (Bragança et al., 2021a; Pinheiro et al., 2023). The BCB test is typically applied to immature COCs immediately after recovery and prior to IVM. However, the proportion of BCB-positive oocytes varies markedly according to donor characteristics. For instance, approximately 29% of oocytes were classified as BCB-positive in prepubertal goats (Rodríguez-González et al., 2002), whereas 19–47% were reported in prepubertal sheep depending on the BCB concentration used (Catalá et al., 2011). In contrast, higher proportions have been reported in adult donor populations, including 66–69% BCB-positive oocytes in slaughterhouse-derived sheep ovaries (Wang et al., 2012) and up to 85% among morphologically normal oocytes from FSH-primed adult goats (Paramio and Izquierdo, 2014). Consistent with this metabolic selection, BCB-positive oocytes generally show higher maturation, cleavage, and blastocyst rates than BCB-negative counterparts (Rodríguez-González et al., 2002; Catalá et al., 2011; Wang et al., 2012). However, although BCB staining enriches the oocyte population for developmental competence, its effect on the total number of embryos produced is less consistent because the procedure reduces the number of oocytes entering the IVEP pipeline.

Despite improvements in selection strategies, IVM remains a major technical bottleneck in small ruminant IVEP. Conventional maturation systems are inherently static and fail to reproduce the dynamic regulatory mechanisms present *in vivo*, including meiotic arrest control, cyclic nucleotide signaling, and cumulus–oocyte metabolic coupling (Paramio and Izquierdo, 2016). As a consequence, nuclear maturation frequently occurs without coordinated cytoplasmic and metabolic remodeling, limiting developmental competence. In addition, culture under supraphysiological oxygen tension can exacerbate oxidative stress, particularly in oocytes with incomplete cytoplasmic maturation (Luvoni et al., 1996; Agarwal et al., 2006).

Recent transcriptomic studies provide molecular evidence of these limitations, demonstrating that oocytes matured *in vitro* exhibit altered signaling pathways, endoplasmic reticulum stress, and disrupted redox regulation compared with *in vivo*-matured counterparts (Cui et al., 2025). Historically, attempts to overcome these deficiencies have relied on increasingly complex IVM supplementation strategies using serum, follicular fluid, antioxidants, or growth factors (Paramio and Izquierdo, 2016; Souza-Fabjan et al., 2023b). Although these approaches may partially improve embryo development, they often reduce reproducibility across laboratories. More recently, alternative strategies have focused on restoring physiological control of meiotic progression. Biphasic maturation systems based on cyclic AMP modulation, such as Capacitation-IMV (CAPA-IVM), delay meiotic resumption and improve coordination between nuclear and cytoplasmic maturation, resulting in increased blastocyst production (Rose et al., 2013; Medina-Chávez et al., 2021; Ferrer-Roda et al., 2024a,b). Taken together, these findings indicate that improving IVEP efficiency in small ruminants will depend less on refining morphological selection criteria and more on developing maturation systems capable of restoring the dynamic regulatory environment that governs oocyte competence *in vivo*.

### In vitro fertilization (IVF)

IVF is another critical step that influences embryo yield and quality in small ruminant IVEP systems. Even when nuclear maturation is achieved during IVM, oocyte populations remain heterogeneous in their ability to regulate fertilization events such as cortical granule exocytosis and the establishment of an effective polyspermy block. In this context, sperm preparation and capacitation procedures are major sources of variability affecting fertilization outcomes. In ovine and caprine IVF systems, spermatozoa are typically prepared using swim-up or density-gradient centrifugation (e.g., Percoll) to enrich for motile sperm. The swim-up method is more commonly applied to fresh semen, whereas density-gradient centrifugation is frequently preferred for frozen–thawed samples due to its superior capacity to recover functionally competent spermatozoa. Density-gradient centrifugation acts as a selective washing technique that separates sperm based on motility and density, yielding highly enriched populations of motile and morphologically normal spermatozoa while effectively removing cellular debris, leukocytes, epithelial cells, bacteria, and immature or abnormal sperm, including those with compromised DNA integrity (Mortimer, 2000; Henkel and Schill, 2003; Souza-Fabjan et al., 2023a). This method allows further selection of sperm according to their kinetic properties and head density, thereby enhancing the overall quality of the recovered fraction (Mortimer, 2000; Henkel and Schill, 2003).

Following sperm selection, fertilization is generally performed in specialized media. Synthetic Oviductal Fluid (SOF) is the medium most commonly used for ovine IVF, whereas Tyrode’s Albumin Lactate Pyruvate (TALP) is widely used as a fertilization medium in caprine IVF systems (Paramio and Izquierdo, 2014; Souza-Fabjan et al., 2023b). These media are typically supplemented with bovine serum albumin and bicarbonate, which support sperm capacitation, together with heparin or related glycosaminoglycans as capacitating agents, similar to fertilization systems commonly used in bovine IVF. In some protocols, additional capacitation-promoting compounds such as penicillamine, hypotaurine, and epinephrine or estrous serum are included to enhance sperm functionality and fertilization rates (Souza-Fabjan et al., 2023b).

Despite these strategies, ovine and caprine spermatozoa remain highly sensitive to handling, cryopreservation, and oxidative stress, which can compromise motility, membrane integrity, and fertilization capacity (Peris et al., 2004; Barbas et al., 2018; Jurado-Campos et al., 2023). Moreover, conventional static IVF systems fail to fully reproduce the dynamic regulatory environment of the oviduct, increasing the incidence of polyspermy commonly reported (Palomo et al., 2010; Romar et al., 2019; Anzalone et al., 2021). Although polyspermy is particularly pronounced in porcine IVF systems, it also represents a recurrent challenge in ovine and caprine IVF, reflecting the difficulty of reproducing the physiological mechanisms that regulate sperm–oocyte interaction within the oviduct. Supplementation with oviductal fluid proteins has been shown to improve monospermic fertilization and IVF efficiency, highlighting the limitations of conventional systems in reproducing physiological gamete interactions (Bragança et al., 2021b).

The use of sex-sorted semen introduces additional technical challenges in small ruminant reproduction. Flow cytometric sorting exposes spermatozoa to mechanical and oxidative stress, which can reduce motility, membrane integrity, and fertilization potential (Hollinshead et al., 2004; de Graaf et al., 2007a,b; Ngcobo et al., 2024). Embryos generated from sorted sperm may also display altered expression of genes involved in DNA methylation, chromatin remodeling, and metabolic regulation (Beilby et al., 2011). Although commercial availability has recently expanded, particularly in dairy goat breeding systems, the practical use of sex-sorted semen in small ruminants remains geographically limited. Importantly, the impact of sex-sorted semen differs between reproductive technologies. In MOET programs relying on artificial insemination, the reduced fertility associated with sorted sperm can directly compromise fertilization rates and embryo yield. In contrast, IVF systems offer greater flexibility in sperm concentration and capacitation conditions, partially compensating for reduced sperm quality. Consequently, while sex-sorted semen introduces biological and technical constraints, its integration may be more compatible with IVEP systems than with MOET-based embryo production.

### In vitro embryo culture (IVC)

IVC represents the final stage of IVEP and plays a central role in determining embryo quality. Conventional culture systems remain largely static and only partially reproduce the dynamic physicochemical environment of the oviduct and uterus (Souza-Fabjan et al., 2023b; Ebrahimi et al., 2025). The development of SOF, formulated to mimic to composition of the OF in energy substrates (lactate-pyruvate-glucose) and salts, enabled ovine embryos to overcome the classical developmental block at the 8–16-cell stage (Tervit et al., 1972) and is now widely used. However, SOF-based media do not reproduce the complex composition of the native OF and rely on fixed nutrient concentrations despite clear stage-specific metabolic requirements during development (Gardner and Lane, 1997; Thompson et al., 2007). Early embryos primarily utilize pyruvate and lactate, whereas glucose uptake increases during compaction and blastulation, and failure to accommodate these shifts can compromise blastocyst quality.

Culture conditions also influence oxidative balance. Atmospheric oxygen tension increases ROS production, whereas reduced oxygen levels (approximately 5–7%) improve blastocyst development, mitochondrial function, and cryotolerance (Thompson, 2000; Leoni et al., 2007; Souza-Fabjan et al., 2023b). Strategies such as somatic cell co-culture or fetal calf serum supplementation may increase embryo yield but introduce variability and may impair embryo quality or postnatal outcomes (Young et al., 1998; Souza-Fabjan et al., 2023b). In ruminants, exposure to serum during early embryo culture has also been associated with abnormal fetal growth and developmental disorders collectively referred to as large offspring syndrome, which was initially described in sheep and later extensively reported in cattle produced by assisted reproductive technologies (Young et al., 1998; Chen et al., 2013). These abnormalities are thought to arise from epigenetic dysregulation and altered metabolic conditions during early embryogenesis.

Consequently, most IVEP systems currently rely on semi-defined SOF-based media supplemented with bovine serum albumin and amino acids to reduce variability and improve reproducibility. Despite these improvements, developmental efficiency remains limited. In small ruminants, blastocyst rates following IVEP typically range from approximately 20–40% of fertilized oocytes, depending on donor characteristics and laboratory conditions (Cognié et al., 2003; Paramio and Izquierdo, 2016; Souza-Fabjan et al., 2023b). Moreover, embryos produced *in vitro* frequently exhibit altered transcriptomic and epigenetic profiles compared with *in vivo*-derived embryos, as demonstrated in ovine models showing differential gene expression and impaired embryo–endometrium interactions, highlighting that current IVC systems still fail to fully replicate the physiological conditions of the reproductive tract (Wei et al., 2016; Zhu et al., 2021).

Together, these limitations suggest that contemporary IVC systems impose a technical ceiling on embryo developmental potential in small ruminant IVEP. Future improvements in embryo culture efficiency will likely depend on the development of dynamic, physiologically relevant culture systems, including sequential media formulations, microfluidic platforms, and oviduct-on-a-chip technologies that better replicate the *in vivo* reproductive environment (Ferraz and Ferronato, 2023). Overall, these emerging approaches highlight the need for next-generation embryo culture systems that integrate dynamic nutrient regulation, physiological oxygen tension, and reproductive tract-derived signals to more accurately recapitulate early embryonic development *in vivo*.

## Logistical and economic challenges

The implementation of IVEP in small ruminants is constrained by logistical and economic barriers that limit its scalability and commercial feasibility. Compared with cattle, IVEP in sheep and goats requires greater technical specialization per embryo produced, resulting in higher operational costs and strong dependence on centralized infrastructure. From a logistical perspective, the technique relies on highly trained personnel for LOPU, anesthesia, gamete handling, and embryo culture. A single LOPU session often requires multiple specialized operators and veterinary anesthesia support, increasing labor requirements compared with MOET (Souza-Fabjan et al., 2014a; Baldassarre, 2021). In addition, IVEP depends on advanced laboratory infrastructure, including surgical facilities, controlled-atmosphere incubators, and dedicated embryo culture laboratories, which restricts its use to specialized centers and limits on-farm application (Paramio and Izquierdo, 2014).

Another economic constraint is the routine use of hormonal stimulation to promote follicular development before LOPU (Souza-Fabjan et al., 2023b; Baldassarre, 2021). In sheep and goats, IVEP typically relies on FSH- or eCG-based protocols to obtain adequate oocyte yields, generating recurring donor-level costs (Baldassarre and Karatzas, 2004). Variability in ovarian response may also require protocol adjustments or repeated stimulation cycles, further increasing hormone use and overall costs (Baldassarre, 2021). As a result, the combined expenses associated with surgical procedures, anesthesia, consumables, hormonal treatments, and specialized labor lead to a relatively high cost per transferable embryo, which is often higher than that of MOET-derived embryos (Baldassarre and Karatzas, 2004). Scalability represents an additional limitation. The invasive nature of LOPU restricts the frequency of oocyte collection from individual donors, while the reliance on laboratory-based workflows limits large-scale application under field conditions. Consequently, IVEP in small ruminants remains largely confined to high-value genetic programs, germplasm conservation, and research applications.

## Emerging perspectives for IVEP in small ruminants

**Figure 5:**
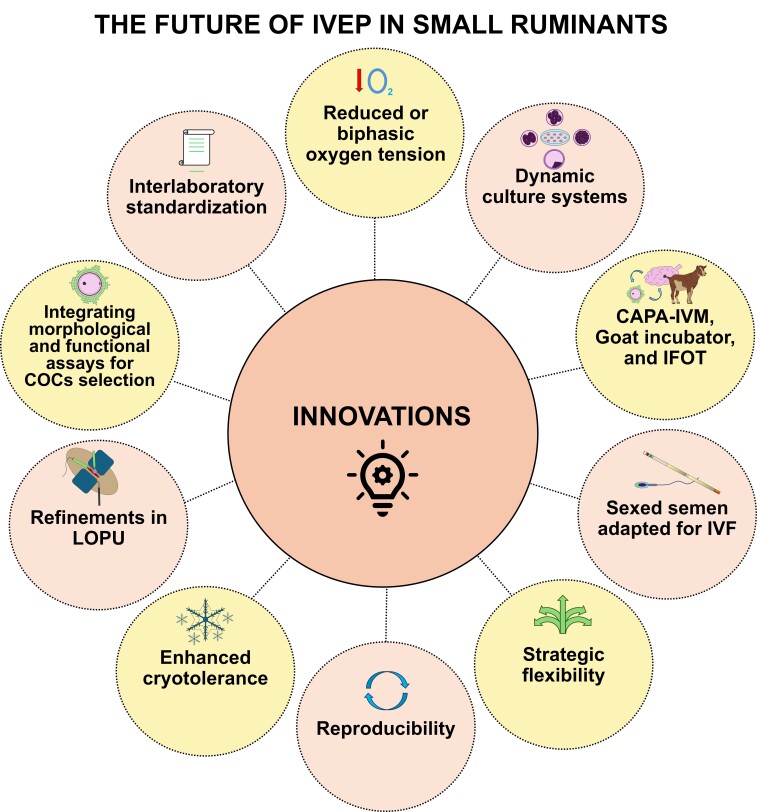
Emerging innovations and research directions aimed at improving *in vitro* embryo production (IVEP) efficiency in small ruminants. Strategies include advances in oocyte competence, fertilization systems, and embryo culture technologies that may enhance embryo yield, cryotolerance, and scalability of IVEP platforms. COCs: cumulus–oocyte complexes; CAPA-IVM: biphasic *in vitro* maturation; IFOT: intrafollicular oocyte transfer; LOPU: laparoscopic ovum pick-up; IVF: *in vitro* fertilization.

Despite decades of methodological refinement, IVEP in small ruminants remains constrained by biological inefficiencies, logistical complexity, and relatively high operational costs, which together limit its scalability and field applicability. These challenges have shifted the research focus from incremental optimization of conventional protocols toward innovative strategies that integrate advances in reproductive biology, cellular metabolism, and bioengineering. Current perspectives increasingly emphasize improving oocyte developmental competence, reducing procedural invasiveness and hormonal dependency, and enhancing system reliability under less restrictive laboratory conditions. The next phase of technological progress will likely depend on translating mechanistic insights into simplified, cost-effective platforms capable of expanding IVF beyond specialized centers (Figure 5).

## What could change the balance?

**Figure 6:**
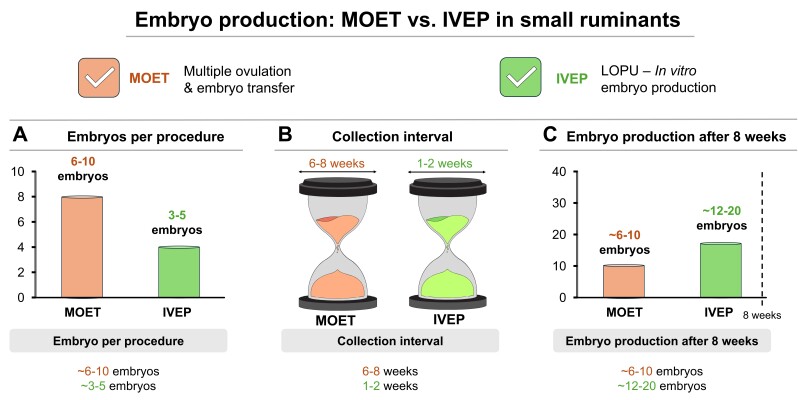
Conceptual comparison between *in vivo* embryo production (MOET) and *in vitro* embryo production (IVEP) systems in small ruminants based on embryo production dynamics. (A) Typical number of embryos obtained per procedure, (B) interval between consecutive collections, and (C) estimated cumulative embryo production over an eight-week period. Although MOET generally produces more embryos per session, the shorter collection interval of IVEP may result in comparable cumulative embryo output over time.

Historically, the balance between MOET and IVEP in small ruminants has been determined not only by biological efficiency but also by operational simplicity, cost–benefit relationships, and robustness under field conditions. Although MOET remains the dominant approach due to its reproducibility and logistical feasibility, advances across the IVEP pipeline suggest that this balance could shift if improvements translate into scalable and economically viable outcomes. Current LOPU–IVEP systems typically recover approximately 10 and 14 viable oocytes per session in sheep and goats, respectively, resulting in 3–5 transferable embryos and pregnancy rates exceeding 50% after embryo transfer (Baldassarre, 2021). Although this embryo yield per session is generally lower than that achieved through MOET, which commonly produces 6–10 transferable embryos per superovulatory cycle, IVEP offers the possibility of shorter intervals between collections, often every 1–2 weeks, compared with the 6–8 week recovery interval typically required for MOET. Consequently, when embryo output is evaluated over a defined period, IVEP may achieve comparable or even greater cumulative embryo production (Gibbons et al., 2007; Souza-Fabjan et al., 2023a) (Figure 6). When pregnancy rates are also considered, the overall number of pregnancies generated per donor over time may approach similar levels between systems under optimized conditions.

One factor contributing to the potential strategic value of IVEP is the increasing availability of sex-sorted semen adapted for IVF. Historically, the use of sexed sperm in sheep and goats has been limited by reduced fertilization rates, increased DNA fragmentation, and altered capacitation dynamics associated with sorting and cryopreservation (Hollinshead et al., 2004; de Graaf et al., 2007b; Bathgate, 2008). However, recent refinements in sperm handling and IVF-specific capacitation protocols have improved fertilization outcomes and blastocyst development (Bai et al., 2025), increasing the strategic value of IVEP in breeding programs where control of offspring sex may be advantageous. For example, the production of female offspring may be preferred in dairy-oriented systems to accelerate genetic gain through replacement females, whereas male offspring may be desirable in specific breeding or terminal-cross programs. Nevertheless, in many sheep and goat breeding schemes, both sexes are economically valuable, and in some programs, sex-sorted semen is not routinely used because both male and female offspring contribute to genetic progress and herd structure. On the female side, refinements in LOPU, including improved aspiration control and follicular synchronization, have increased oocyte recovery and procedural consistency (Teixeira et al., 2011; Baldassarre, 2021). Nevertheless, oocyte quality remains a major limitation, and integrating morphological and functional selection methods, such as BCB staining, can enrich oocyte populations with greater developmental potential (Rodríguez-González et al., 2003; Catala et al., 2011; Alm et al., 2020).

Advances in embryo culture systems may also improve IVEP outcomes. Studies in bovine and human models indicate that reduced oxygen tension, dynamic culture systems, and supplementation with bioactive components can enhance blastocyst quality and post-cryopreservation survival (Chen et al., 2023; Alegretti et al., 2024; Báez et al., 2024). Similarly, extracellular vesicles derived from reproductive fluids have been shown to improve embryo cryosurvival by modulating lipid metabolism and redox balance (Leal et al., 2022). These findings highlight embryo cryotolerance as a critical determinant of IVEP competitiveness, a domain in which MOET-derived embryos continue to hold a practical advantage. However, translating these strategies to ovine and caprine systems remains challenging due to species-specific differences in embryo metabolism and oxidative sensitivity. Without rigorous species-specific validation and cost–benefit evaluation, improvements in laboratory performance may not substantially alter the economic balance between MOET and IVEP.

Global embryo production data reported by the IETS in 2024 clearly reflect these constraints, where MOET-based production still accounted for more than 95% of ovine embryos worldwide. Although ovine IVEP increased substantially between 2022 and 2023 (+62%), *in vivo* production also expanded (+79%), suggesting overall market growth rather than technological replacement. In goats, IVEP activity also increased but remains geographically concentrated. Importantly, this pattern is not consistently observed in the 2025 dataset, largely due to a reduction in the number of reporting countries, which limits direct comparisons and likely underestimates global embryo production trends. Integration with genomic selection may represent one of the most transformative applications of advanced reproductive technologies in small ruminants. By enabling earlier and more precise identification of superior donors and sires, genomic information can improve the efficiency of both MOET and IVEP programs. In this context, IVEP may provide additional advantages by allowing repeated oocyte recovery and rapid multiplication of genetically superior females, thereby increasing selection intensity and shortening generation intervals (Kasinathan et al., 2015; Meuwissen et al., 2016). Ultimately, a shift in the balance between MOET and IVEP will depend on the convergence of improved biological robustness, embryo cryotolerance, economic scalability, and alignment with breeding objectives.

## Realistic expectations

Despite significant technological progress, the expansion of IVEP in small ruminants must be interpreted within realistic biological, logistical, and economic constraints. Many advances that could theoretically shift the balance between IVEP and MOET remain limited by challenges related to cryotolerance, reproducibility, and scalability. Consequently, current evidence suggests that IVEP is more likely to expand as a strategic complementary tool rather than replace MOET as the dominant reproductive technology in sheep and goat production systems. MOET remains technically simpler, more convincing under field conditions, and economically predictable for large-scale application, particularly in extensive and semi-intensive systems. In contrast, IVEP depends on specialized infrastructure, skilled personnel, and tightly controlled laboratory environments, restricting its routine use primarily to nucleus herds, research institutions, and high-value breeding programs. Under these circumstances, IVEP is best positioned to complement MOET in situations where *in vivo* embryo production is limited. Such scenarios include the use of prepubertal donors, animals with reduced reproductive performance, or individuals under intense genetic selection. IVEP also offers strategic advantages when integrated with sexed semen, genomic selection, and intensive breeding schemes designed to accelerate genetic gain, even when total embryo output remains lower than that achievable with MOET. In this context, IVEP functions as a precision-oriented rather than volume-oriented reproductive technology.

Future progress will likely depend on approaches that simultaneously improve oocyte competence, embryo cryotolerance, and the robustness of simplified culture systems. Additional knowledge on peri-conception in small ruminants and derived potential innovations, including non-invasive embryo assessment, dynamic culture environments, and improved metabolic support during early development, may further expand the applicability of IVEP. However, the long-term impact of these technologies will ultimately depend on their economic feasibility and compatibility with production systems. When strategically integrated, IVEP can complement MOET and enhance the flexibility and genetic efficiency of advanced breeding programs without fundamentally replacing existing reproductive frameworks.

## Conclusions

Multiple ovulation and embryo transfer remains the backbone of embryo production in small ruminants, largely due to its technical simplicity (when NSER is applied), robustness under field conditions, and predictable cost–benefit ratio across diverse production systems. Despite significant technological advances, IVEP has not yet achieved the operational scalability required to replace MOET as the dominant reproductive technology. Nevertheless, IVEP has undergone substantial biological and technical refinement over the past decade. Improvements in oocyte recovery, fertilization protocols, embryo culture systems, and the integration of sexed semen and genomic tools have increased its strategic value, particularly in high-value breeding contexts. These advances position IVEP as a precision-oriented technology capable of addressing specific limitations of MOET rather than functioning as a direct substitute.

Looking forward, the most realistic trajectory for IVEP lies in its targeted integration within advanced breeding programs. Future progress will depend less on maximizing blastocyst yield and more on improving embryo cryotolerance, system reproducibility, interlaboratory standardization, and economic viability under field conditions. When aligned with clearly defined breeding objectives—such as accelerating genetic gain or disseminating elite female genetics—IVEP has the potential to complement MOET and enhance the flexibility of reproductive management systems in small ruminants. Ultimately, the future of assisted reproduction in these species will likely depend not on replacing existing technologies but on strategically integrating complementary approaches that combine biological efficiency with practical applicability across diverse production environments.

## Data Availability

No research data was used.
